# Integrated analyses of multi-omics reveal global patterns of methylation and hydroxymethylation and screen the tumor suppressive roles of HADHB in colorectal cancer

**DOI:** 10.1186/s13148-018-0458-3

**Published:** 2018-03-02

**Authors:** Yimin Zhu, Hanlin Lu, Dandan Zhang, Meiyan Li, Xiaohui Sun, Ledong Wan, Dan Yu, Yiping Tian, Hongchuan Jin, Aifen Lin, Fei Gao, Maode Lai

**Affiliations:** 1 Department of Epidemiology and Biostatistics, School of Public Health, Zhejiang University, Hangzhou, 310058 China; 2 BGI-Shenzhen, Shenzhen, 518083 China; 3 Agricultural Genomics Institute at Shenzhen, Chinese Academy of Agricultural Sciences, Shenzhen, 518120 China; 4 Key Laboratory of Disease Proteomics of Zhejiang Province and Department of Pathology, School of Medicine, Zhejiang University, Hangzhou, 310058 China; 5 Laboratory of Cancer Biology, Provincial Key Lab of Biotherapy in Zhejiang, Sir Runrun Shaw Hospital, Medical School of Zhejiang University, Hangzhou, China; 6 Human Tissue Bank/Medical Research Center, Taizhou Hospital of Zhejiang Province, Wenzhou Medical University, Linhai, Zhejiang 317000 China; 7 Department of Pathology, School of Medicine, Zhejiang University, 866 Yuhangtang Road, Zhejiang, Hangzhou 310058 China

**Keywords:** DNA methylation, DNA hydroxymethylation, Sequencing, Colorectal cancer, Epigenetic

## Abstract

**Background:**

DNA methylation is an important epigenetic modification, associated with gene expression. 5-Methylcytosine and 5-hydroxymethylcytosine are two epigenetic hallmarks that maintain the equilibrium of epigenetic reprogramming. Disequilibrium in genomic methylation leads to carcinogenesis. The purpose of this study was to elucidate the epigenetic mechanisms of DNA methylation and hydroxymethylation in the carcinogenesis of colorectal cancer.

**Methods:**

Genome-wide patterns of DNA methylation and hydroxymethylation in six paired colorectal tumor tissues and corresponding normal tissues were determined using immunoprecipitation and sequencing. Transcriptional expression was determined by RNA sequencing (RNA-Seq). Groupwise differential methylation regions (DMR), differential hydroxymethylation regions (DhMR), and differentially expressed gene (DEG) regions were identified. Epigenetic biomarkers were screened by integrating DMR, DhMR, and DEGs and confirmed using functional analysis.

**Results:**

We identified a genome-wide distinct hydroxymethylation pattern that could be used as an epigenetic biomarker for clearly differentiating colorectal tumor tissues from normal tissues. We identified 59,249 DMRs, 187,172 DhMRs, and 948 DEGs by comparing between tumors and normal tissues. After cross-matching genes containing DMRs or DhMRs with DEGs, we screened seven genes that were aberrantly regulated by DNA methylation in tumors. Furthermore, hypermethylation of the HADHB gene was persistently found to be correlated with downregulation of its transcription in colorectal cancer (CRC). These findings were confirmed in other patients of colorectal cancer. Tumor functional analysis indicated that HADHB reduced cancer cell migration and invasiveness. These findings suggested its possible role as a tumor suppressor gene (TSG).

**Conclusion:**

This study reveals the global patterns of methylation and hydroxymethylation in CRC. Several CRC-associated genes were screened with multi-omic analysis. Aberrant methylation and hydroxymethylation were found to be in the carcinogenesis of CRC.

**Electronic supplementary material:**

The online version of this article (10.1186/s13148-018-0458-3) contains supplementary material, which is available to authorized users.

## Background

Cancer is a disease driven by the accumulation of genetic mutations [1] and the disruption of epigenetic regulation [2]. Epigenetic modifications are associated with gene expression [3, 4]. DNA methylation, such as methylation of cytosine to 5-methylcytosine (5-mC), is catalyzed de novo and maintained by DNA methyltransferases (DNMTs) [5, 6], and this methylation is preserved through cell division [7, 8]. The aberrant regulation of DNA methylation, such as global hypomethylation or regional hypermethylation, has consistently reported as an important epigenetic hallmark of cancers, including colorectal cancer [8, 9]. For example, hypermethylation of CpG islands in the promoter regions and in exon 1 represses or even silences the transcriptional expression of tumor suppressor genes (TSG) and promotes carcinogenesis.

In addition to DNA methylation, DNA hydroxymethylation (5-hmC) is another important epigenetic hallmark for cancers. 5-hmC is synthesized from 5-mC by ten-eleven translocation (TET) proteins [10, 11]. TET proteins further oxidize 5-hmC into 5-formylcytosine and 5-carboxylcytosine. An unmethylated cytosine is restored by the removal of the carboxyl group from 5-carboxylcytosine by the enzyme thymine-DNA glycosylase (TDG). Therefore, 5-hmC is regarded as an intermediate during active demethylation and is believed to help maintain the equilibrium of epigenetic reprogramming [12–16]. Despite this, 5-hmC has been observed as a stable epigenetic modification, especially in the cancer genome, where reduced levels have been previously reported [17, 18].

Although there is a significant amount of data regarding the global distribution of 5-mC in colorectal cancer, there is a great need for examining both 5-mC and 5-hmC simultaneously. Because of their resistance to bisulfite conversion, 5mC and 5hmC cannot be distinguished from each other using only bisulfite sequencing data [19]. In order to understand the role of DNA demethylation, a series of techniques have been developed to accurately differentiate cytosine methylation states, including hMeDIP-seq, oxBS-seq, and TAB-seq [14, 20, 21]. Compared to enrichment steps, methods like oxBS-seq and TAB-seq require an immense amount of sequencing and are very costly. In the present study, we collected tumors and the corresponding adjacent normal tissues from six colorectal cancer patients, then determined the levels of genome-wide DNA methylation by methylated DNA immune-precipitation sequencing (MeDIP-seq) and hydroxymethylation by hydroxymethylated DNA immunoprecipitation sequencing (hMeDIP-seq). Their transcriptional expression was determined using RNA-seq. We found a distinct genome-wide hydroxymethylation pattern that could be used as an epigenetic biomarker for differentiating colorectal tumor tissues from normal tissues. Furthermore, hypermethylation of the hydroxyacyl-CoA dehydrogenase trifunctional multi-enzyme complex subunit beta gene (HADHB) was persistently found to be correlated with its transcriptional downregulation in colorectal cancer (CRC). The differences in methylation, hydroxymethylation, and transcriptional expression of HADHB between cancerous and normal tissues were confirmed in additional colorectal cancer patients. To further validate these findings, we performed functional analyses and found that the overexpression of HADHB clearly reduced cancer cell migration and invasiveness. These results suggest that HADHB could play the role of a TSG. In brief, this study provided valuable data for the screening of epigenetic biomarkers and for elucidating the epigenetic mechanisms of carcinogenesis in colorectal cancer.

## Methods

### Tissue collection and preparation

Colorectal tumor samples, as well as the corresponding adjacent normal tissues (5 cm away from the edge of the tumor), were surgically collected and then preserved in liquid nitrogen. The genomic DNA and RNA of each sample were extracted using Qiagen’s DNA and RNA extraction kits, respectively. The study protocols were approved by the research ethics committees of Zhejiang University School of Medicine (2012-1-012) and BGI-Shenzhen (NO. BGI-IRB 15060). All participants signed the written informed consent form.

### Library construction and data analysis of RNA-seq

The total RNA samples were first treated with DNase I to degrade any possible DNA contamination. The mRNA was then enriched using oligo (dT) magnetic beads and mixed with a fragmentation buffer to be fragmented into approximately 200-bp fragments. First-strand cDNA synthesis was performed using random hexamers. Buffer, dNTPs, RNase H, and DNA polymerase I were added to synthesize the second strand. The double-stranded cDNA was purified with magnetic beads. End preparation and 3′-end addition of the nucleotide adenine (A) were performed. Finally, sequencing adaptors were ligated to the fragments. The fragments were enriched by PCR amplification. During the QC step, the Agilent 2100 Bioanalyzer and ABI StepOnePlus Real-Time PCR System were used to qualify and quantify the DNA library. The library products were then sequenced with the Illumina HiSeq 2000.

The levels of gene expression level and the differentially expressed genes were analyzed using the method described by Audic and Claverie [22]. Levels of gene expression were calculated using the reads per kilobase million (RPKM) method. In cases where more than one transcript was found for a gene, the longest read was used to calculate its expression level and coverage. The RPKM values were then used directly to compare gene expression differences between the tumor and the normal samples. The significantly differentially expressed genes (DEG) were determined at a threshold false discovery rate (FDR) ≤ 0.05 and the absolute value of log2ratio ≥ 0.585.

### Library construction and data analysis of MeDIP-seq and hMeDIP-seq

Prior to immunoprecipitation, 5 μg of genomic DNA was sonicated to a mean fragment size of 200 bp, followed by end repair with the addition of deoxyadenosine (dA) and adaptor ligation, according to the Illumina Paired-End protocol. MeDIP-Seq and hMeDIP-Seq libraries were constructed, as described in a previous study [23]. The libraries were sequenced using the Illumina HiSeq analyzer, according to the manufacturer’s instructions. After base calling, low-quality reads were omitted, and the clean reads were aligned to the UCSC human reference genome hg19 using SOAP2 (Version 2.21). Mismatches of no more than two bases were allowed in the alignment.

### Identification of DMR and DhMR between tumors and corresponding normal tissues

Identification of groupwise differential methylation regions (DMR) and differential hydroxyl-methylation regions (DhMR) was performed using a sliding windows strategy along the entire genome, as described in our previous study [24]. This strategy identified DMR and DhMR between tumors and the corresponding normal tissues, based on a threshold of *P* < 0.05 and at least five CG sites.

### Functional enrichment analysis for DMRs and DhMR in promoters

Functional enrichment analysis was performed by Gene Ontology (GO) and pathway analysis using the DAVID (Database for Annotation, Visualization, and Integrated Discovery) web server (http://david.abcc.ncifcrf.gov). Genes with DMRs, DhMR in promoters, and DEG were mapped to their respective human orthologs, and the lists were submitted to DAVID for enrichment analysis to determine any significant overrepresentation of GO biological processes (GO-BP), molecular functions (GO-MF), and KEGG-pathway categories. For all analyses, the known, full-length genes were set as the background, and the *P* values (EASE score), indicating the significance of the overlap between various gene sets, were calculated using Benjamini-corrected modified Fisher’s exact test. Only GO-BP, GO-MF, or KEGG-pathway terms with *P* values less than 0.05 were considered significant and listed as differentially expressed.

### Quantitative PCR of HADHB expression

Total RNA was isolated from cells using TRIzol (Invitrogen, USA). The concentration of RNAs was measured and normalized using a spectrophotometer (Eppendorf, Hamburg, Germany). Reverse transcription was performed using a PrimeScript RT reagent kit (Perfect Real Time) and real-time PCR was performed using the SYBR Premix Ex Taq (Tli RNaseH Plus), both from TaKaRa Biotechnology Co., Ltd. (Dalian, China). The following PCR primers were used to amplify HADHB: 5′- ACACTGTCACCATGGCTTGT -3′ (forward) and 5′- CTGGCCAGAAGCAATCAAG -3′ (reverse). For GAPDH, the following primers were used: 5′-ACCACAGTCCATGCCATCAC-3′ (forward) and 5′-TCCACCACCCTGTTGCTG TA-3′ (reverse). GAPDH was used as the reference gene. The Ct values of the samples were calculated, and the relative levels of HADHB mRNA were analyzed by the 2^−△△Ct^ method.

### Cell culture and plasmid construction

Human colorectal carcinoma cell lines, HT29 and HCT8, were obtained from the American Type Culture Collection (ATCC). HT29 and HCT8 were maintained in liquid nitrogen and incubated in 5% CO_2_ at 37 °C in a PRMI1640 medium with 10% fetal bovine serum (FBS).

The pcDNA3.1 (+) vector was sliced using restriction enzymes Xhol1 and Bamh1. First-strand cDNA was synthesized using the HiScript® 1st Strand cDNA Synthesis Kit (Vazyme biotech co., Ltd., Suzhou, China). The complete coding sequence of HADHB was PCR-amplified with the following primers: 5″–CTTGGTACCGAGCTCGGATCCATGAC TATCTTGACTTACCCCTTTAAAA–3′ (forward) and 5′–CCCTCTAGATGCATGCTCG A GTTATTTTGGATAAGCTTCCACTATCAT–3″ (reverse). The PCR product was then inserted into the linearized pcDNA3.1 to perform recombination cloning using the ClonExpress II one-step cloning kit (Vazyme Biotech Co., Ltd.). The recombined products were verified by DNA sequencing and transfected using Lipfectamine™2000, according to the manufacturer’s instructions. The efficiency of overexpression was validated using qRT-PCR and western blot analyses.

### RNA interference analysis

Small interfering RNA (siRNA) against HADHB and a negative control siRNA were purchased from Shanghai Genepharma Co. Ltd. (Shanghai, China). The anti-HADHB siRNA sequence was 5`-GCACAGUGACAGCUGCAAATT-3`, which was not homologous to any other human DNA sequence. HT29 and HCT8 cells were cultured in six-well plates in antibiotic-free DMEM for 48 h and transfected using the PowerFect™ siRNA Transfection Reagent (SigmaGen) according to the manufacturer’s instructions. The efficiency of knockdown was determined by qRT-PCR and western blot analyses.

### Western blot analysis

Cells were extracted using a RIPA lysis buffer and prepared according to the standard procedure. Proteins were extracted using 12% SDS-PAGE and transferred onto nitrocellulose membranes. The membranes were blocked with 5% skimmed milk in TBS-Tween 20 for 2 h before being incubated overnight with primary antibodies at 4 °C. They were then incubated with secondary antibodies at 20 °C for nearly 1 h. After extensive washing in TBST, the protein level was measured using the Odyssey system (Li-COR, Lincoln, NE, USA). The loading was monitored by GAPDH. Primary antibodies were directed against HADHB (Abcam, 1:1000) and GAPDH (Santa Cruz, CA, USA, 1:5000).

### Cell counting kit-8 (CCK-8) assay

Cells were plated in 96-well plates (1 × 10^3^ cells/well) with 100 μl of the medium. The absorbance at 450 nm was measured to estimate the relative number of viable cells after culturing with 10 μl of CCK-8 reagent, which was purchased from Boster Biological Technology Co., Ltd. (Wuhan, China). The analysis was performed in three replicate wells for each sample and repeated for three times.

### Cell migration and invasion assays

Transwell 24-well Boyden chambers (8-μm pores; Costar, Corning, NK, USA) were used to measure cell migration and invasion according to the manufacturer’s protocol. For studying migration, cells (1 × 10^5^) in 200 μl of a serum-free medium were seeded on the upper chamber, and 600 μl of a complete medium containing 10% FBS was added to the lower chamber as a chemoattractant. After incubation at 37 °C for 20 h (HCT8 cells) and 96 h (HT29 cells), the non-migratory cells were removed with cotton swabs. Cells on the lower surface of the membrane were fixed in 4% paraformaldehyde and stained with crystal violet solution. The number of invading cells was counted in five randomly selected fields using an inverted microscope equipped with a digital camera at × 40 magnification.

For the cell invasion assay, 2 × 10^5^ cells were seeded in the upper chamber, which had been coated with 50 μL Matrigel basement membrane matrix (BD Biosciences, USA), followed by incubation for 16 h (HCT8 cells) and 96 h (HT29 cells).

## Results

### Generation and characterization of CRC methylome and hydroxymethylome

Six patients with colorectal cancer, three with rectal cancer, and three with colon cancer were recruited for this study. The characteristics of these patients are summarized in Additional file 1: Table S1, including gender, age, and pathological types. Primary tumor tissue samples and their adjacent normal tissues were collected after surgery. We applied MeDIP-seq and hMeDIP-seq technologies to examine whole-genome DNA methylation and hydroxymethylation patterns, respectively, for all 12 DNA samples. MeDIP-seq and hMeDIP-seq technologies allow for the highly efficient enrichment of methylated and hydroxymethylated DNA fragments [25] using antibodies against methylated and hydroxymethylated cytosines, respectively. On average, 120.1 and 117.9 million paired-end reads, 50 bp in length, were generated from MeDIP-seq and hMeDIP-seq, respectively. Of these reads, 116.0 (96.63%) and 114.2 (96.88%) million clean reads were aligned to the human reference genome hg19. After removing the ambiguously mapped reads, we acquired 99.7 (83.03%) million and 104.7 (87.32%) million uniquely aligned reads, reaching an average depth coverage of 3.49 and 3.71 for DNA methylation and hydroxymethylation, respectively (Additional file 1: Table S2 and Additional file 2: Figure S1). To enable pair-wise comparisons across different samples, we used reads per million (RPM) as a measure of the methylation and hydroxymethylation levels in a genomic region in order to normalize the data.

**Fig. 1:**
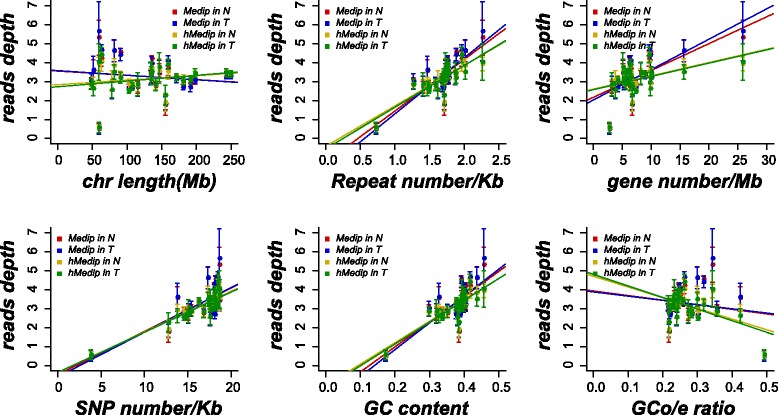
Global patterns of methylome and hydroxymethylome. The Pearson’s correlation between DNA methylation and hydroxymethlation levels and features of autosomes (chromosomes 1–12) and sex chromosomes X and Y. The read depth was plotted against the length, repeat density, gene density, SNP density, GC content, and CpGo/e ratio of the individual chromosome. The line represents linear regression. Different colors represent the different modifications in tumor and normal tissues (red is Medip in normal tissue, blue is Medip in tumor tissue, gold is hMedip in normal tissue, and green is hMedip in tumor tissue)

We first characterized the global patterns of methylome and hydroxymethylome by correlating their read depths with the number of different genomic elements. In general, both DNA methylation and hydroxymethylation were positively correlated with the number of repeat sequences, gene number, SNP number, and GC content, both in the tumors and adjacent normal tissues (Fig. 1). No significant correlation was found between chromosome length and ratio of observed and expected number of CpGs (CpG O/E), although similar patterns of methylation and hydroxymethylation were observed in relation to GC content. High levels of hydroxymethylation and methylation were found in the regions of high GC content of approximately 50 to 60% (Additional file 3: Figure S2). Furthermore, uneven distribution of methylation and hydroxymethylation was found in the features of chromosomes in tumors and normal tissues, especially at transcriptional start sites (TSS) and CpG islands (CGIs), where there were lower levels. In contrast, CGI shores (regions that flank CGIs with less CG density) showed higher levels of methylation and hydroxymethylation than other genomic elements. The highest level of methylation, but not hydroxymethylation, was observed in short interspersed elements (SINEs), which are highly repetitive sequences (Additional file 4: Figure S3). These findings indicated that the distribution of both 5-mC and 5-hmC was closely dependent on the characteristics of the genomic sequences (Fig. 1, Additional file 1: Table S3), which were consistent with previous studies [23, 25].

### Positive correlation between global methylation and hydroxymethylation levels

The whole genome was divided into 0.5-kb windows, and the levels of 5-mC and 5-hmC were classified into different groups, according to the RPMs of MeDIP and hMeDIP, respectively. The correlations between methylation and hydroxymethylation are shown in Additional file 5: Figure S4. The levels of methylation were positively correlated with those of hydroxymethylation in tumors (Pearson’s correlation coefficient *r* = 0.9630, *P* = 2.843e−12) and normal tissues (Pearson’s correlation coefficient *r* = 0.9686, *P* = 6.115e−13). These results were consistent with previous results from mouse hippocampus [26], human brain [27], and pancreatic cancer [28]. As 5-hmC is believed to be an intermediate compound in the oxidation reaction of 5-mC, this finding suggests that methylated regions may be constantly undergoing reprogramming, depending on the cell type. For different cell populations, the hotspot of epigenomic reprogramming may vary. For instance, in neurons and stem cells, 5-mC usually co-localizes with heterochromatin, whereas 5-hmC co-localizes with euchromatin [29–31]. In the current CRC study, we found that, when comparing tumors with normal tissues (slope of fitted line = 0.234), the gradient response of hydroxymethylation against methylation was lower in tumor tissues (0.178) (Additional file 5: Figure S4). This result suggested that tumor tissues in CRC display a global reduction in 5-hmC compared to normal tissues like the majority of cancers [32–35].

### Distinct global pattern of hydroxymethylome, but not methylome, in CRC

In addition to the correlation coefficient difference between 5-mC and 5-hmC, we also observed variations in the average levels of the two types of DNA modifications, when comparing tumors and normal tissues. For instance, compared to normal tissues, tumor tissues showed higher than average levels of DNA methylation in TSSs, promoters, exons, transcriptional end sites (TES), CpG islands, CGI shores, and SINEs, but lower than average hydroxymethylation levels in exons, introns, gene bodies, SINEs, TESs, enhancers, and CGI shores (Additional file 4: Figure S3). Additionally, higher inter-individual variations in tumors were suggested for methylation and hydroxymethylation levels in nearby TSSs and TES [28], as indicated by the comparisons of standard deviations (SD) between tumors and normal tissues (*P* < 0.001, paired *t* test) (Additional file 6: Figure S5). These results suggested that the potential dysregulation of epigenetic modifications could lead to large-scale latent instability, which might cause carcinogenesis.

**Fig. 2:**
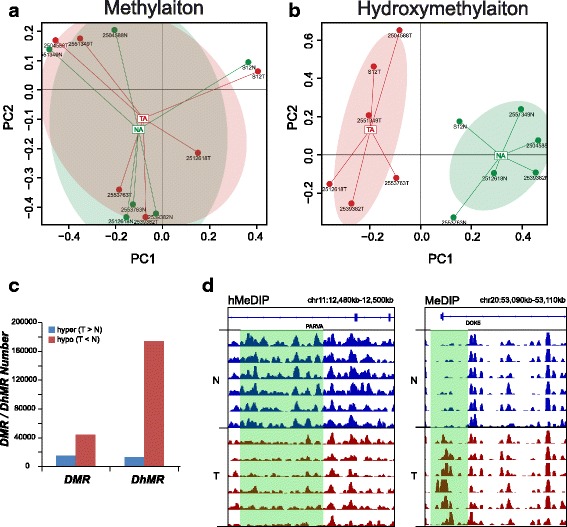
Different distributions of methylation and hydroxymethylation. **a**, **b** Principal component (PC) analysis based on methylation and hydroxymethylation levels for 0.5-kb tiles across 6 normal and tumor tissue samples. Coloring indicates classification of samples into subgroups. The red area denotes tumor tissues and the green area means normal tissue. **c** A large number of regions of differential hydroxymethylation (DhMRs) and methylation (DMRs) occur in colorectal cancer. Regions that gain a mark (“hyper-”) are represented by blue bars, whereas losses (“hypo-”) are red. **d** Visualization of DMRs (left) and DhMRs (right) patterns across all normal and tumor tissues (blue and red bars in the box). Boxed regions with green are candidate DMRs or DhMRs

Based on these observations, we used principal component analysis to infer the inter-group global patterns of methylome and hydroxymethylome, using the RPM of 0.5-kb windows across the whole genome. The global methylation pattern of tumor tissues and normal tissues could not be clearly differentiated (Fig. 2a). In contrast, a clear separation between the tumors and normal tissues was observed in the principal component analysis (PCA) of hydroxymethylation, indicating distinct patterns of the hydroxymethylome in tumors, compared to normal tissues (Fig. 2b). Therefore, a global change in the hydroxymethylome can be considered as a key characteristic of CRC. Gilat et al. also found that global levels of 5-hmC could distinguish between colon tumors and normal colon tissue adjacent to the tumor based on the levels [36]. Bhattacharyya et al. [28] reported a similar discovery in pancreatic cancer, in which they found that the distribution pattern of 5-hmC samples were strikingly different from those of normal cells.

### Pair-wise comparison revealing extensive DhMRs and DMRs in CRC

Next, we applied a sliding-window strategy to identify differentially methylated regions (DMRs) and differentially hydroxymethylated regions (DhMRs) in tumors and normal corresponding normal tissues, in order to reveal key genomic regions with significant DNA methylation and hydroxymethylation changes during carcinogenesis. Based on the threshold of *P* < 0.05 and at least five CG sites, we obtained 59,249 DMRs and 187,172 DhMRs (Fig. 2c). The representative differential regions of DMR (Fig. 2d, right) and DhMRs (Fig. 2d, left) were presented. Most DMRs and DhMRs were more frequently distributed (observed/expected ratio > 1) in promoter regions, exons, enhancers, and repeat sequences, such as LTRs, LINEs, and SINEs. Unlike DMRs, DhMRs were also frequently distributed in TES regions (Additional file 7: Figure S6). Aberrant methylations or hydroxymethylations in promoter regions were less frequent than those in other regions, consistent with previous observations in cancer [25]. Despite this, aberrant DNA modifications within promoters were most likely correlated with altered gene expressions [37, 38]. Therefore, we provided further annotation to the genes with DMRs or DhMRs. Because the gene promoter is the most important regulatory element in the genome and the aberration of methylation and hydroxymethylation in this region may be associated with carcinogenesis, we focused on the genes with DMRs and/or DhMRs in the promoter. We obtained 1699 and 7864 genes containing DMRs and DhMRs in the promoter, respectively. The lists of these genes are presented in Additional file 1: Table S4 and Table S5, respectively. KEGG analysis was performed with the WebGestalt tool (http://www.webgestalt.org). We found 49 significant pathways enriched in genes containing DMRs and 170 containing DhMRs, respectively. The top five methylation-enriched functional pathways were neuroactive ligand-receptor interactions, tight junctions, pathways in cancer, long-term depression, and Chagas disease. Pathways with enriched DhMRs included glycosylphosphatidylinositol (GPI)-anchor biosynthesis, African trypanosomiasis (sleeping sickness), tyrosine metabolism, tryptophan metabolism, and shigellosis (Additional file 1: Table S6).

### CRC transcriptome profiling reveals epigenetics-regulated gene expression changes

We also performed RNA-seq to determine the transcriptome-wide changes in CRC, compared to adjacent normal tissues. We obtained 14.0 to 19.2 million reads per sample, of which 96.5 to 99.2% were clean data. Most of these clean reads (91.4% - 94.1%) could be uniquely aligned to the human reference genome hg19, and 45.0–61.2% were mapped to RefSeq genes (Additional file 1: Table S7). The expression levels were measured in terms of reads per kilobase per million (RPKM) and were used to further analysis. Based on a strict threshold (FDR-adjusted *P* < 0.05 and fold change > 1.5 in four or more samples), 948 significant DEGs were identified between the cancer and the normal samples (Additional file 1: Table S8). From these 948 genes, 12 KEGG pathways were found to be enriched, from which the following top five categories were found to be relevant to tumorigenesis: cell cycle, purine metabolism, metabolic pathways, ribosome biogenesis in eukaryotes, and ribosomes (Additional file 1: Table S6). We also cross-matched these DEG KEGG pathways with those previously identified to be enriched in DMR- and DhMR-containing genes; we found that metabolic pathways, purine metabolism, and axon guidance were shared features in these groups (Additional file 8: Figure S7).

**Fig. 3:**
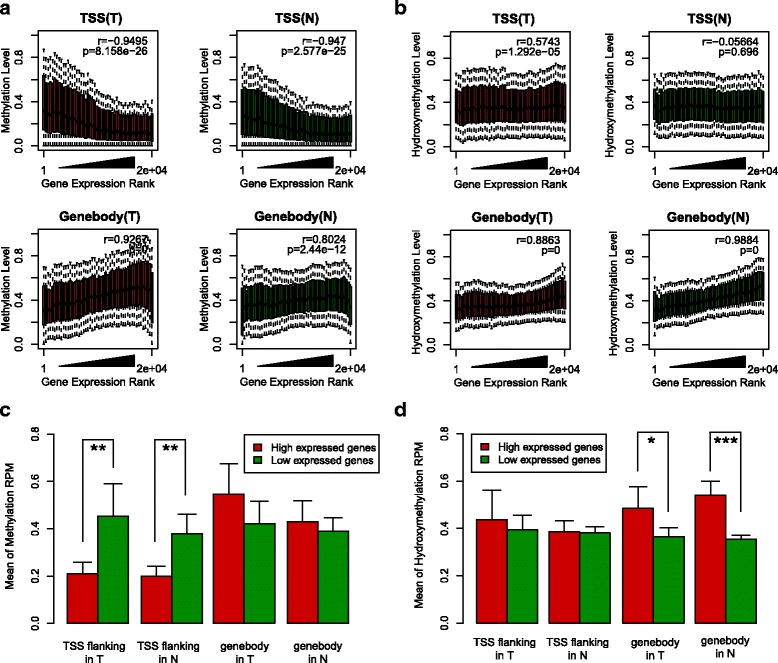
Association between epigenomic modification and gene expression. **a**, **b** Correlations between epigenomic modification (**a** is 5-mC, **b** is 5-hmC) and gene expression in TSS (defined as − 500 bp to + 500 bp across TSS) and genebody respectively. For Medip, there is a clear anti-correlation of gene expression in TSS and a positive correlation in the genebody. For hMedip, only positive correlations in the genebody can be observed. **c** 5-mC is enriched in the TSS flanking region (defined as − 500 bp to + 500 bp across TSS) of lowly expressed genes in both despite normal and tumor tissues. **d** 5-hmC is enriched in the genebody region of highly expressed genes in both normal and tumor tissues

We then correlated the expression levels of all genes with levels of methylation or hydroxymethlation in their promoter regions (TSS ± 500 bp) and gene bodies (Fig. 3a, b). Gene expression levels in tumors and normal tissues were negatively correlated with promoter methylation, but positively correlated with gene body methylation, which has been reported in previous genome-wide analyses [28, 39]. A similar positive correlation was observed for hydroxymethylation in the gene body, although no clear correlation was observed in promoter regions. When we classified the associated genes into genes with high and low expression levels, the highly expressed genes displayed significantly lower levels of promoter methylation, but significantly higher levels of hydroxymethylation in the gene body (Fig. 3c, d). These results suggest that many DEGs could be potentially regulated by promoter methylation or gene body hydroxymethylation.

### Integrated analyses identifying DEGs aberrantly regulated by DNA methylation in tumors

In order to identify the DEGs that were aberrantly regulated by DNA modifications in tumors, we cross-matched the genes containing DMRs or DhMRs with DEGs. Considering the role of 5-hmC as an intermediate of demethylation, we reasoned that the genes with hypermethylation and hypohydroxymethylation in promoters would be most stably repressed within a cell population, while genes with hypomethylation and hyper-hydroxymethylation in promoters would have a greater chance of being expressed. With this reasoning, we identified seven genes that contained both DMRs and DhMRs. These seven genes were HIGD1A, AHCYL2, IL11RA, CHL1, SEMA6D, BIRC3, and HADHB (Additional file 1: Table S9). Among these genes, the methylation of HIGD1A and CHL1 has been reported in common tumors. Expressions of SEMA6D, IL11RA, and BIRC3 genes have been reported to be associated with tumors; however, no association between colorectal cancer and methylation has been reported. There have been no reports on tumors or methylation associated with HADHB or AHCYL2 genes. Thus, we are the first to report associations between HADHB and AHCYL2 genes and tumors and between the methylation of SEMA6D, IL11RA, and BIRC3 genes and tumors.

### HADHB as a potential tumor suppressor gene aberrantly repressed by promoter hypermethylation in CRC

**Fig. 4:**
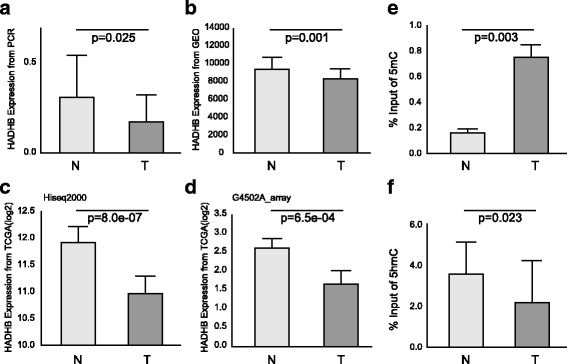
Expression and epigenetic modification validation of HADHB in additional samples and public functional genomics data. **a** Expression level of HADHB determined by real-time PCR in 15 additional pairs of colorectal tumors and their adjacent normal tissues. **b** Expression data of HADHB from the GEO database. **c**, **d** Expression level of HADHB from the TCGA database. The results derived from different platform (**c** is Hiseq200 and **d** is G450 array) are shown respectively. **e**, **f**, Methylation and hydroxymethylation levels were determined by real-time PCR in 15 additional pairs of colorectal tumors and their adjacent normal tissues

Importantly, we identified one DEG, HADHB, which showed hypermethylation and hypohydroxymethylation with significantly downregulated expression in CRC (Additional file 9: Figure S8). To confirm that the expression level of the HADHB gene was associated with its methylation and hydroxymethylation levels in a larger population, we collected 15 additional pairs of samples from colorectal tumors and their adjacent normal tissues. The methylation and hydroxymethylation levels were determined with MeDIP and hMeDIP, respectively, followed by real-time PCR to determine the expression level of HADHB (Additional file 6). We also collected the expression data for HADHB from The Cancer Genome Atlas (TCGA) (http://cancergenome.nih.gov/) and the Gene Expression Omnibus (GEO) (http://www.ncbi.nlm.nih.gov/geo/) databases. We found the expression levels of HADHB in tumor tissues (0.18 ± 0.15) were significantly lower than those in normal tissues (0.32 ± 0.24) (*P* = 0.025) (Fig. 4a). This result was consistent with the findings from data of GEO (Fig. 4b), TCGA sequencing (Fig. 4c), and TCGA array (Fig. 4d). Compared to normal tissues, tumor tissues had higher levels of methylation (0.74 ± 0.19 vs 0.17 ± 0.068) (Fig. 4e) and lower levels of hydroxymethylation (2.58 ± 1.97 vs 3.48 ± 1.52) (Fig. 4f). These results indicate that lower levels of HADHB expression in tumor tissues are associated with higher levels of methylation and lower levels of hydroxymethylation. This is consistent with the results of genome-wide sequencing analyses of methylation and hydroxymethylation. The HADHB gene may be a potential tumor suppressor gene, whose expression is modified by methylation and reduced by hydroxymethylation.

**Fig. 5:**
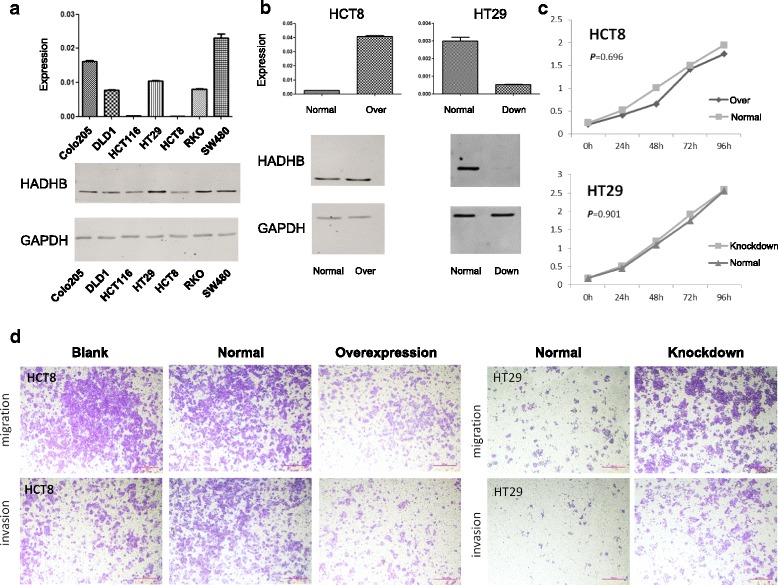
Functional experiments of HADHB on CRC cell line. **a** Transcriptional and translational levels of HADHB in seven cell lines detected by RT-PCR and western blot. **b** HADHB expression in knockdown and overexpression cell lines of HT29 and HCT8. **c** Cell growth curves in the knockdown and overexpression cell lines of HT29 and HCT8. **d** Cells migration and invasion in the knockdown and overexpression cell lines of HT29 and HCT8

To evaluate the potential contribution of the HADHB gene to tumorigenesis, we further performed gene knockdown and overexpression experiments in colorectal cancer cell lines. After evaluating the expression levels of HADHB in seven cell lines using reverse transcriptase PCR (RT-PCR) and western blot analyses, we selected the HCT-8 cell line for HADHB overexpression and the HT-29 cell line for HADHB knockdown, in which HADHB expression was the lowest and highest, respectively (Fig. 5a). Our results showed that the expression of HADHB efficiently decreased in the HADHB-knockdown HT-29 cell line and increased in the HADHB-overexpressed HT-8 cell line, compared with that in the normal cell lines (Fig. 5b). In the subsequent characterization of cell capacity, we found that knockdown and overexpression of HADHB had no effect on cell growth (Fig. 5c). Interestingly, the migration and invasion of cells were significantly reduced in cells overexpressing HADHB. In contrast, HADHB knockdown caused enhanced migration and invasion (Fig. 5d).

Taken together, these functional experiments support the hypothesis that HADHB is a potential tumor suppressor gene, which can reduce tumor cell invasiveness and migration, suggesting that silencing HADHB may contribute to colorectal oncogenesis and progression.

## Discussion

DNA methylation has become a promising biological marker of cancer risk, diagnosis, and prognosis. DNA methylation in the promoter can repress or silence gene expression. Therefore, 5-mC is usually considered the “fifth base” of DNA. The discovery of hydroxymethylation, often considered to be the sixth base of DNA, has increased the complexity of methylation research. While 5-hmC is mostly believed to be an intermediate of the demethylation process catalyzed by the TET enzyme, many studies have shown that the TET enzyme and 5-hmC act as regulatory factors. Therefore, genome-wide analyses of DNA methylation and hydroxymethylation, which are important epigenetic biomarkers, will help reveal aberrantly regulated tumor suppressor genes that may be involved in carcinogenesis.

Bisulfite treatment-based sequencing technologies cannot distinguish between these two types of epigenetic modifications. Methods like oxBS-seq and TAB-seq require an immense amount of sequencing and are costly. Instead, immunoprecipitation by methylation and hydroxymethylation-specific antibodies, combined with next-generation sequencing, can be used to determine genome-wide methylation and hydroxymethylation. In this study, we applied MeDIP-seq, hMeDIP-seq, and RNA-seq for a thorough screening of the epigenome and transcriptome of colorectal tumors.

Previous studies have revealed that aberrant methylation and hydroxymethylation in cancers occur in either specific or global genomic regions [2, 8, 15]. Therefore, by comparing the distribution of methylation and hydroxymethylation between tumors and normal tissues, this study provides valuable data for screening epigenetic biomarkers. Our results showed distinct hydroxymethylome, but not methylome, global methylation patterns in CRCs and their adjacent normal tissues, using PCA. Specifically, divergent hypohydroxymethylation regions were more often located in gene bodies, TESs, enhancers, LTRs, LINEs, and SINEs. Overall, genomic methylation correlated with hydroxymethylation. However, these two modifications do not usually coexist on the DNA [25, 31]. 5-mC usually co-localizes in the heterochromatin, whereas 5-hmC has been found to co-localize in the euchromatin [29, 30]. It can be inferred that euchromatin-specific conversion of 5-mC to 5-hmC is regulated by a combination of cell cycle-dependent chromatin decompensation events and Tet enzyme instability [29]. Our genome-wide study suggested that 5-hmC is a stable potential predictive biomarker for CRC.

Although we did not observe distinct global methylation patterns, we did observe a higher frequency of divergently methylated regions, when comparing tumors and normal tissues, specifically in CGIs, promoters, exons, enhancers, LTRs, LINEs, and SINEs. Previous studies have confirmed that promoter hypermethylation might cause reduced gene expression and contribute to carcinogenesis, including colorectal cancer [25, 39, 40]. Based on genome-wide, pair-wise comparative analyses of tumors and the corresponding normal tissues, 170 significant pathways were found to be enriched in 7864 genes with DhMRs, 49 pathways in 1699 genes with DMRs, and 12 pathways in 948 DEGs. Among these pathways, metabolic pathways, purine metabolism, and axon guidance were overlapped. This suggests that these pathways may be involved in the carcinogenesis of colorectal cancer.

By linking the divergent regions with gene expression, we identified 26 DEGs with both DMRs and DhMRs. Seven genes in which DEGs contained both DMRs and DhMRs were identified (Additional file 1: Table S9): HADHB, HIGD1A, AHCYL2, IL11RA, CHL1, SEMA6D, and BIRC3. The methylation of five genes (AHCYL2, IL11RA, SEMA6D, BIRC3, and HADHB) was associated with tumors, and four genes (IL11RA, CHL1, SEMA6D, and BIRC3) were associated with colorectal cancer. HIGD1A, hypoxia-inducible gene domain 1A, is a mitochondrial protein and a positive regulator of cytochrome c oxidase, which is regulated by hypoxia-inducible factor-1α (HIF1α) [41]. During glucose deprivation, HIGD1A regulates oxygen consumption, ROS production, and AMPK activity to modulate cell survival and tumor growth [41]. The promoter of the HIGD1A gene is differentially methylated in human cancers, preventing its hypoxic induction. This protein is also a potential marker of metabolic stress in vivo and is frequently observed in diverse pathological states such as myocardial infarction, hypoxic-ischemic encephalopathy (HIE), and different cancers [42, 43]. AHCYL2 (adenosyl-homocysteinase like 2) is highly homologous to IRBIT, which regulates ion-transporting proteins. It may also be a potential regulator of NBCe1-B in mammalian cells [44]. However, its function remains unclear. CHL1 (cell adhesion molecule), which encodes a member of the L1 family of neural cell adhesion molecules, is essential for brain development and is involved in signal transduction pathways. It has been found to play an important role in carcinogenesis and cancer progression. He et al. had found that CHL1 was downregulated in human breast cancer and was associated with lower-grade tumors [45]. This downregulation is mediated by DNA methylation. Therefore, CHL1 may be a putative tumor suppressor gene in breast cancer and other common cancers [45–47]. Interleukin 11 receptor subunit alpha (IL11RA), a stromal cell-derived cytokine, is overexpressed in patients with human osteosarcoma and advanced breast cancer with bone metastasis. Additionally, amplification was detected at 9p13.3, where the IL11RA gene is located. Some primary gastric adenocarcinoma samples (19.1%) were found to have an increased copy number of IL11RA [48]. Semaphorin 6D (SEMA6D) has been previously implicated in immune responses, heart development, and neurogenesis. SEMA6D has been reported to be highly expressed in vascular epithelial cells in gastric cancer; it was also positively correlated with the expression of vascular endothelial growth factor receptor 2 (VEGFR2). Therefore, SEMA6D may be associated with tumor angiogenesis [49, 50]. The HADHB gene encodes the beta subunit of a mitochondrial trifunctional protein that catalyzes the last three steps of mitochondrial beta-oxidation of long-chain fatty acids. Mutations in the HADHB gene have been associated with mitochondrial trifunctional protein deficiency [51, 52]. HADHB interacts with estrogen receptor alpha and affects beta-oxidation activity [53]. Hypermethylation in the HADHB gene was found in hepatocellular carcinoma [54]. The baculoviral IAP repeat, which contains the 3 apoptosis inhibitor 2 (BIRC3) and encodes a member of the IAP family of proteins, has multi-biological functions. It not only regulates caspases and apoptosis, but also modulates inflammatory signaling and immunity, mitogenic kinase signaling and cell proliferation, and cell invasion and metastasis. Overexpression of BIRC3 is associated with glioma progression and aggression and chronic and acute B cell lymphocytic leukemia [55, 56]; it is also a predictor of therapeutic resistance to treatment with irradiation, doxorubicin, and temozolomide [57, 58].

In this study, we used a two-stage strategy to confirm hypermethylation and hypohydroxymethylation of the promoter region of the HADHB gene, which exhibited significantly decreased expression in CRC. The functional studies indicated that HADHB might act as a tumor suppressor gene. Therefore, our findings implicated HADHB as a potential biomarker for the diagnosis and treatment of CRC, especially for alleviating and controlling cancer progression.

## Conclusions

To summarize, this study characterized global patterns of methylome and hydroxymethylome and found a genome-wide distinct hydroxymethylation pattern that could be used to differentiate between tumor tissues and normal tissues. We screened 59,249 DMRs, 187,172 DhMRs, and 948 significant DEGs. After integrating genome-wide expression with genome-wide patterns of DNA methylation and hydroxymethylation, we identified 7 genes that were aberrantly regulated by DNA methylation in tumors and were possibly associated with carcinogenesis of colorectal cancer. We confirmed that HADHB could be a novel tumor suppressor gene.

## Additional files

Additional file 1: **Table S1**. The basic characteristics of six patients of colorectal cancer. **Table S2**. Summary of MeDIP-seq and hMeDIP-seq data production. **Table S3**. Pearson's product-moment correlation for the levels of DNA methylation and DNA hydroxymethylation with the elements in chromosome. **Table S4**. Differentially methylated regions (DMRs) in promoters. **Table S5**. Differentially hydroxymethylated regions (DhMRs) in promoters. **Table S6**. The top 10 pathways enriched in DMRs, DhMRs and DEG. **Table S7**. Summary of RNA-seq data production. **Table S8**. Differentially expressed genes (DEGs) in 6 pairs of samples. **Table S9**. The list of 7 genes of which DEGs contained both DMRs and DhMRs. (XLSX 2853 kb)

Additional file 2: **Figure S1.** Coverage and depth of reads for MeDIP-seq and hMeDIP-seq. CpG sites coverage with different sequencing depth are presented. MeDIP-seq data are shown at the top and hMeDIP-seq data are shown at the bottom. Different colors indicate tumor and normal samples. (PDF 192 kb)

Additional file 3: **Figure S2.** Methylation and hydroxymethylation levels distribution against GC content(%) and CpG o/e ratio. Higher levels of hydroxymethylation were found in the regions of GC content around 55% - 65% and CpG O/E ratio of 1.14-1.15. The regions with a GC content around 45% - 55% had a higher methylation level. (PDF 271 kb)

Additional file 4: **Figure S3.** Methylation and hydroxymethylation levels distribution in different types of genomic elements. Normal is shown by green, and tumor is shown by red. TSS: transcriptional start sites; TES: transcriptional end sites; CGI: CpG islands; LTR: long terminal repeat; SINE: short interspersed nuclear elements; LINE: long interspersed nuclear elements. *, *P* < 0.05, **, *P* < 0.01 and ***, *P* < 0.001. (PDF 388 kb)

Additional file 5: **Figure S4.** Correlation between 5-mC and 5-hmC in normal (left) and tumor (right) samples. The whole genome was divided into 0.5 kb windows and the levels of 5-mC and 5-hmC were classified into different groups according to the RPMs of Medip and hMedip, respectively. (PDF 172 kb)

Additional file 6: **Figure S5.** Mean profiles with standard deviation (SD) over TSS and TES regions. 4-kb regions were divided in 80 bins from 5′ to 3′ end, and the mean RPM values (with SD) within each bin for each modification type was determined (5-mC left, 5-hmC right). Additionally, SD distribution are be shown at bottom left in each region. (PDF 217 kb)

Additional file 7: **Figure S6.** Distribution of DMRs and DhMRs in different types of genomic elements. (PDF 162 kb)

Additional file 8: **Figure S7.** Gene functional pathways of DMR, DhMR and DEG. Three pathway, metabolic pathways, purine metabolism and axon guidance, overlapped pathways among DMR, DhMR and DEG. (PDF 138 kb)

Additional file 9: **Figure S8.** Visualization of DMRs (bottom) and DhMRs (top) in HADHB. DMR and DhMR are denoted by the green box. (PDF 231 kb)

## Abbreviations

5-hmC: Hydroxyl-methylation
5-mC: 5-Methylcytosine
CGI: CpG islands
CRC: Colorectal cancer
DEG: Differentially expressed genes
DhMR: Differential hydroxyl-methylation regions
DMR: Differential methylation regions
DNMT: DNA methyltransferase
FDR: False discovery rate
hMeDIP-seq: Hydroxyl-methylation by hydroxymethylated DNA immunoprecipitation sequencing
LINE: Long interspersed nuclear elements
LTR: Long terminal repeat
MeDIP-seq: Methylated DNA immune-precipitation sequencing
RPKM: Reads per kilobase million
SINE: Short interspersed nuclear elements
TDG: Thymine-DNA glycosylase
TES: Transcriptional end sites
TET: Ten-eleven translocation
TSG: Tumor suppressor gene
TSS: Transcriptional start sites
