# Supplementary figures and images for: Integrated analyses of multi-omics reveal global patterns of methylation and hydroxymethylation and screen the tumor suppressive roles of HADHB in colorectal cancer
Source: Clin Epigenetics. 2018 Mar 2;10:30. doi: 10.1186/s13148-018-0458-3 (PMC5833094; doi:10.1186/s13148-018-0458-3)

## 2504588 MeDIP

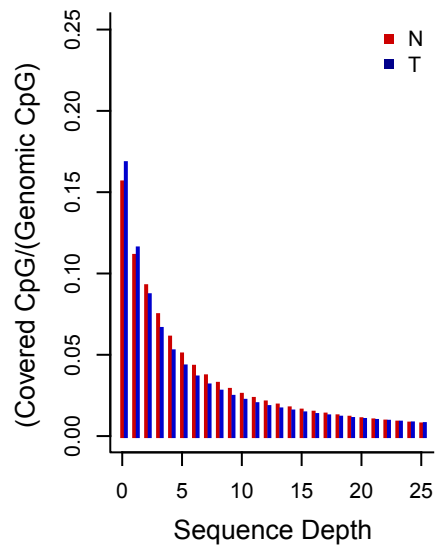

**2512618 MeDIP**

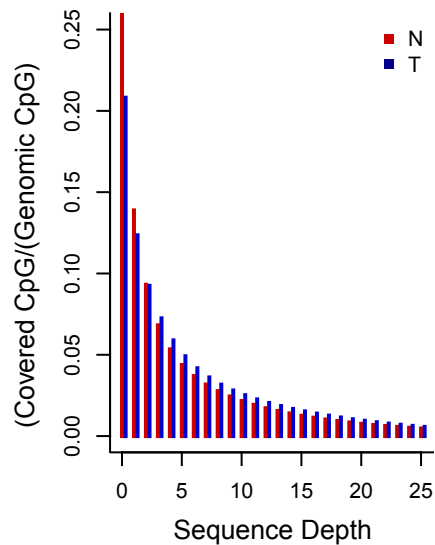

## 2539382 MeDIP

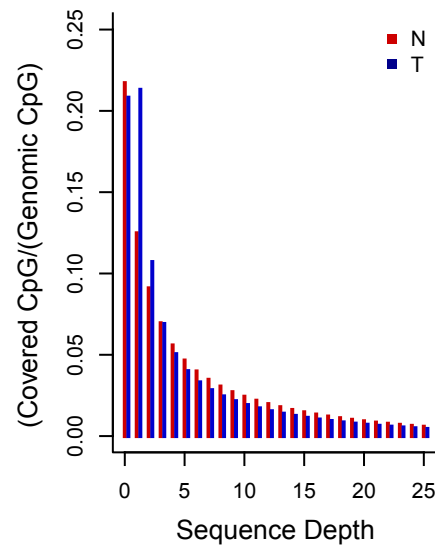

## 2551349 MeDIP

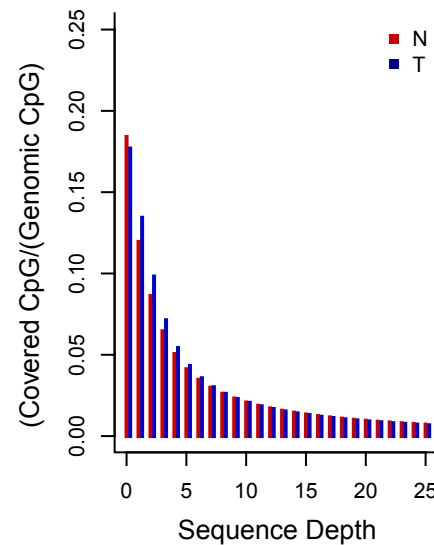

**2553763 MeDIP**

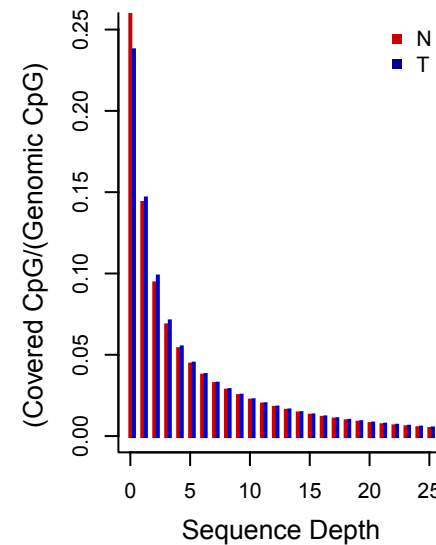

## S12 MeDIP

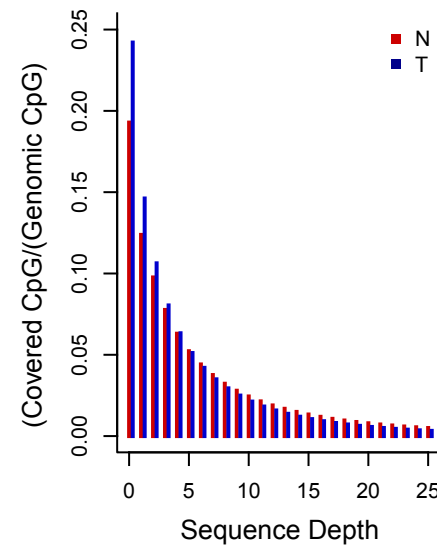

## 2504588 hMeDIP

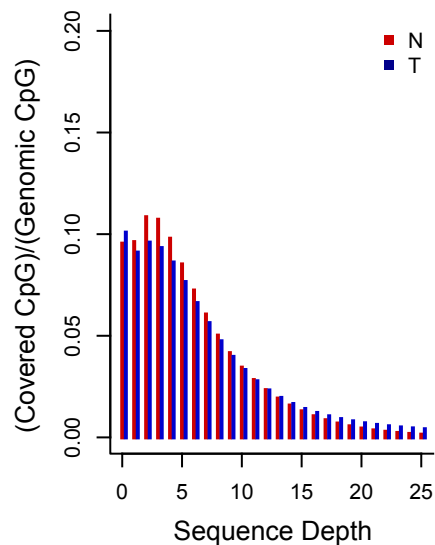

## 2512618 hMeDIP

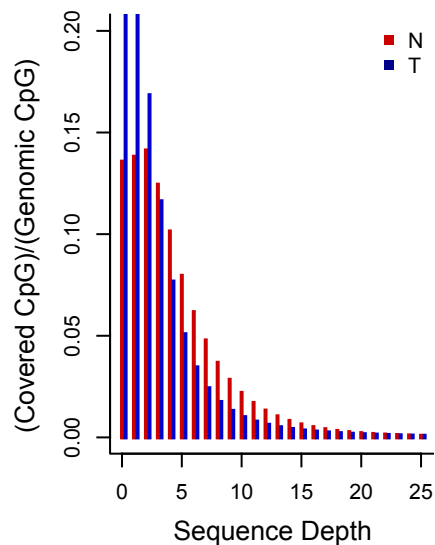

## 2539382 hMeDIP

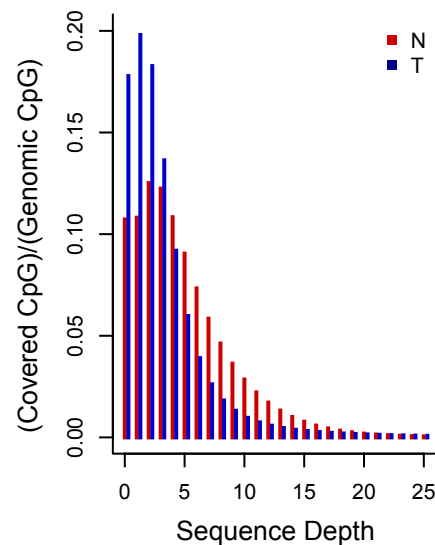

## 2551349 hMeDIP

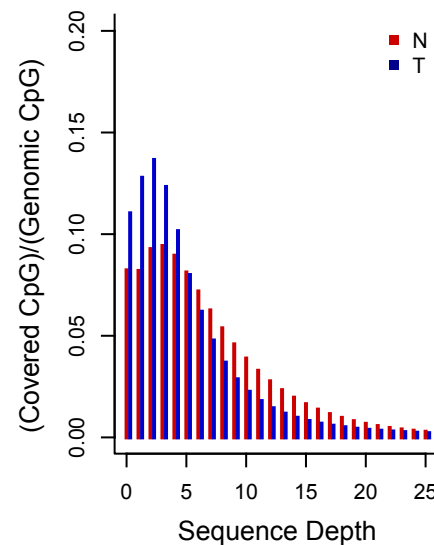

**2553763 hMeDIP**

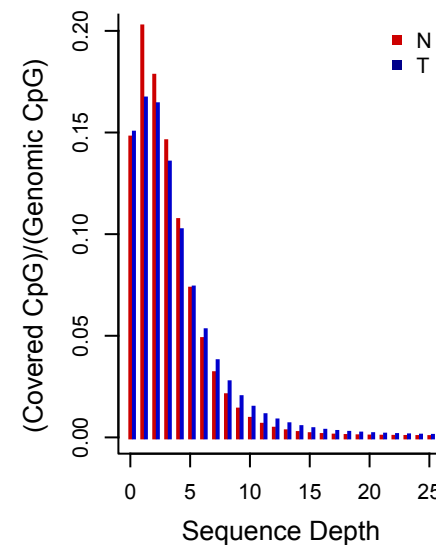

## S12 hMeDIP

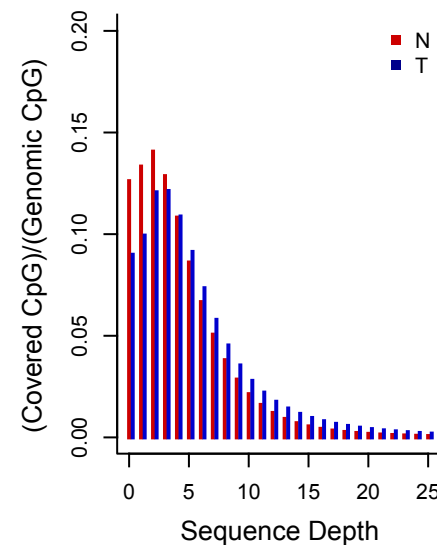

Supplement: Supplementary file 2 — Figure S1. Coverage and depth of reads for MeDIP-seq and hMeDIP-seq. CpG sites coverage with different sequencing depth are presented. MeDIP-seq data are shown at the top and hMeDIP-seq data are shown at the bottom. Different colors indicate tumor and normal samples. (PDF 192 kb) [file 13148_2018_458_MOESM2_ESM.pdf]

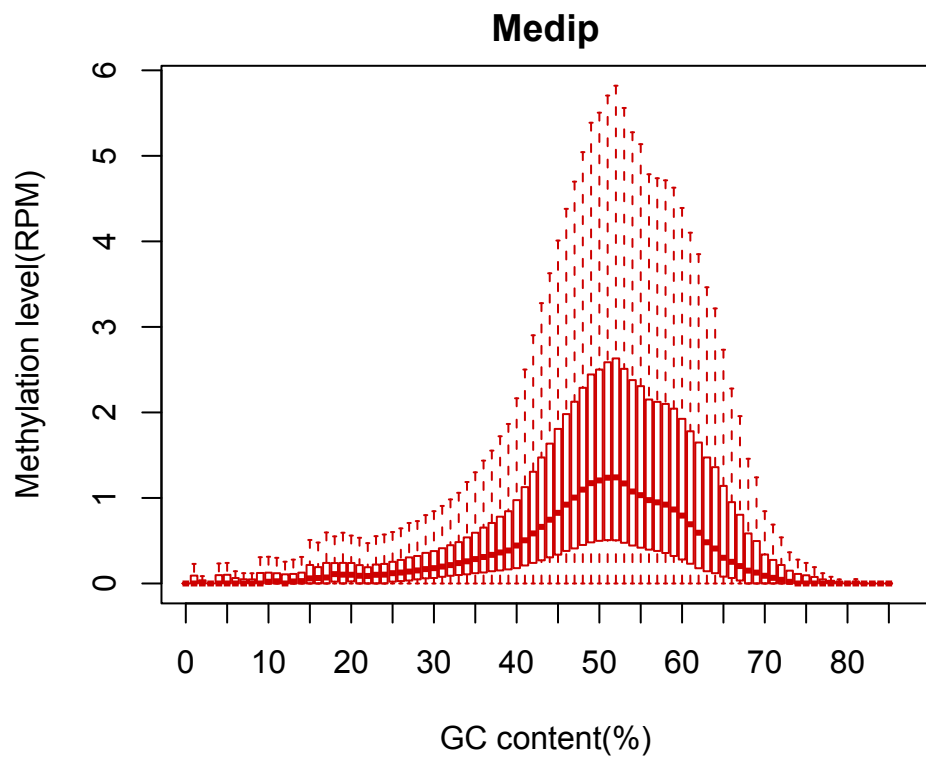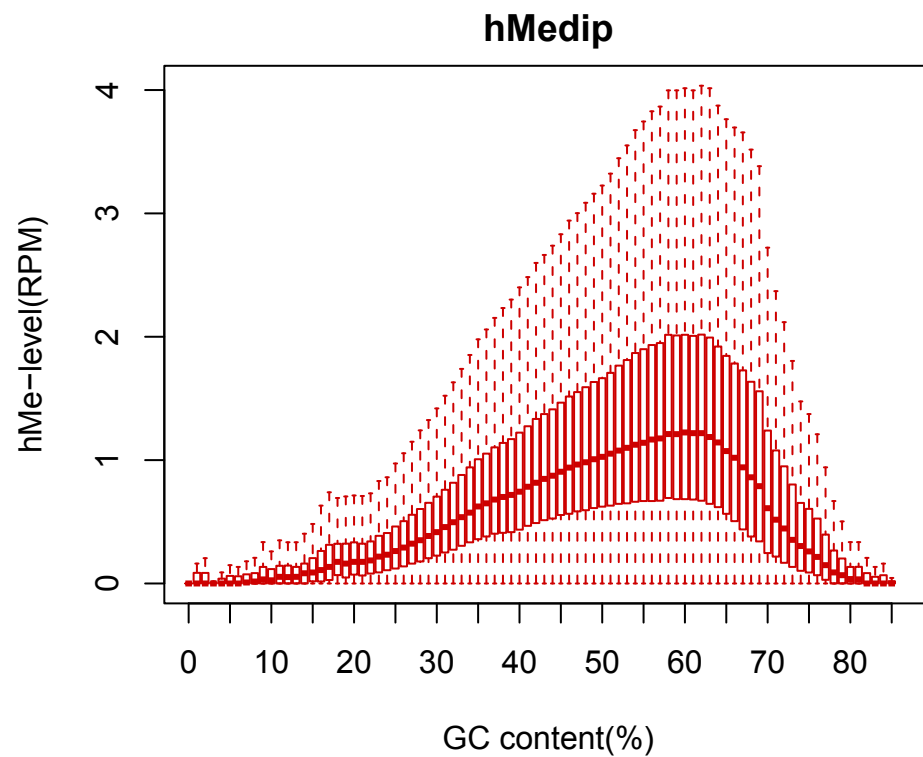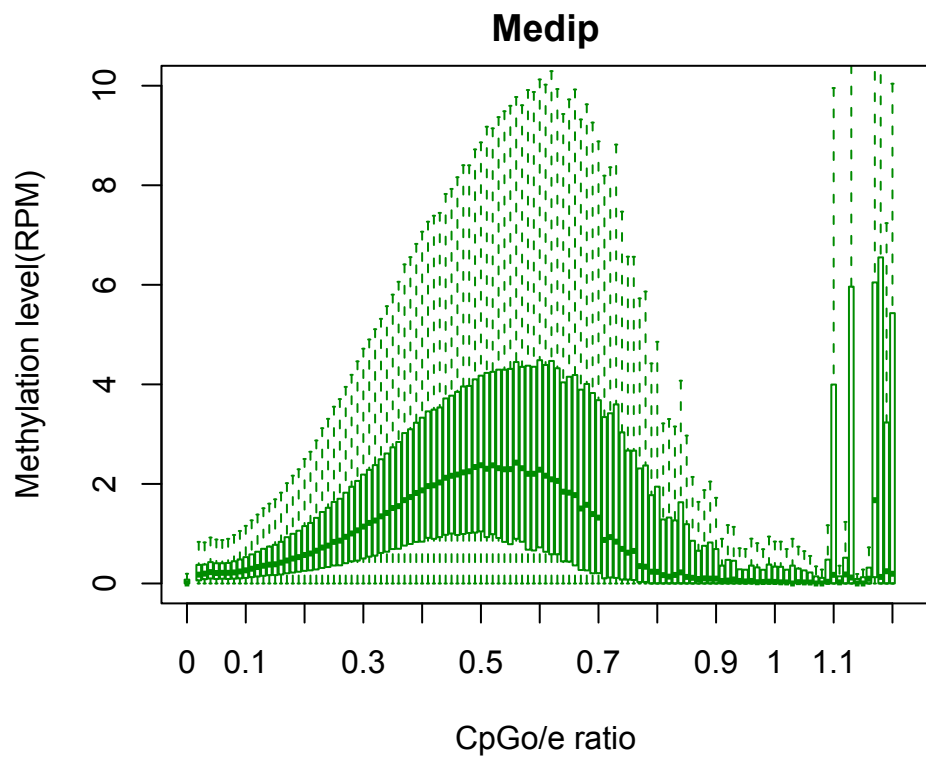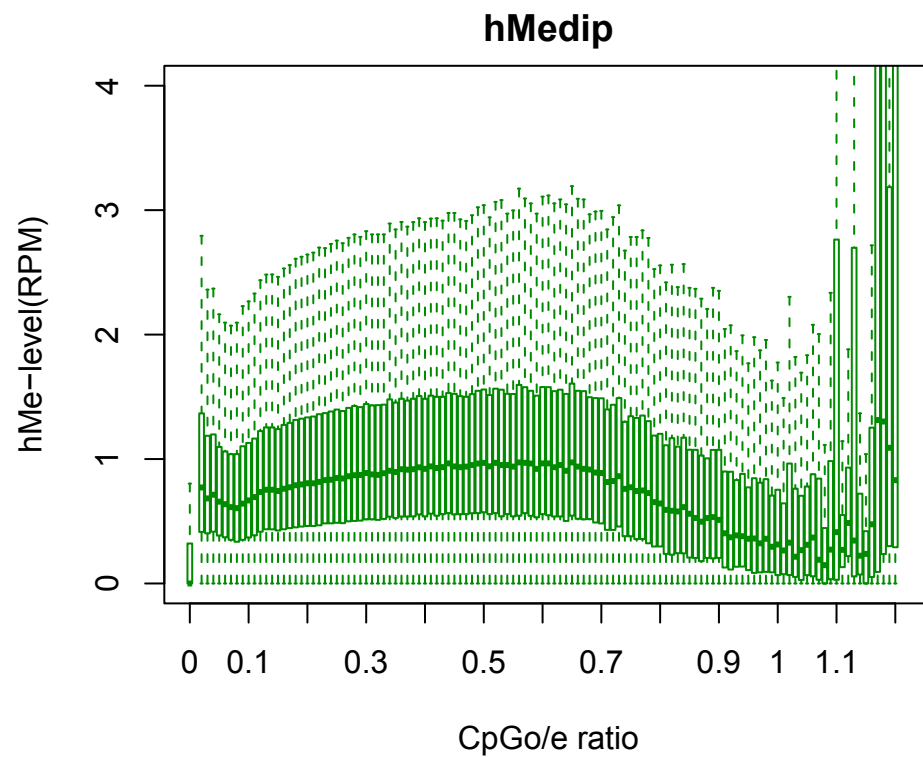

Supplement: Supplementary file 3 — Figure S2. Methylation and hydroxymethylation levels distribution against GC content(%) and CpG o/e ratio. Higher levels of hydroxymethylation were found in the regions of GC content around 55% - 65% and CpG O/E ratio of 1.14-1.15. The regions with a GC content around 45% - 55% had a higher methylation level. (PDF 271 kb) [file 13148_2018_458_MOESM3_ESM.pdf]

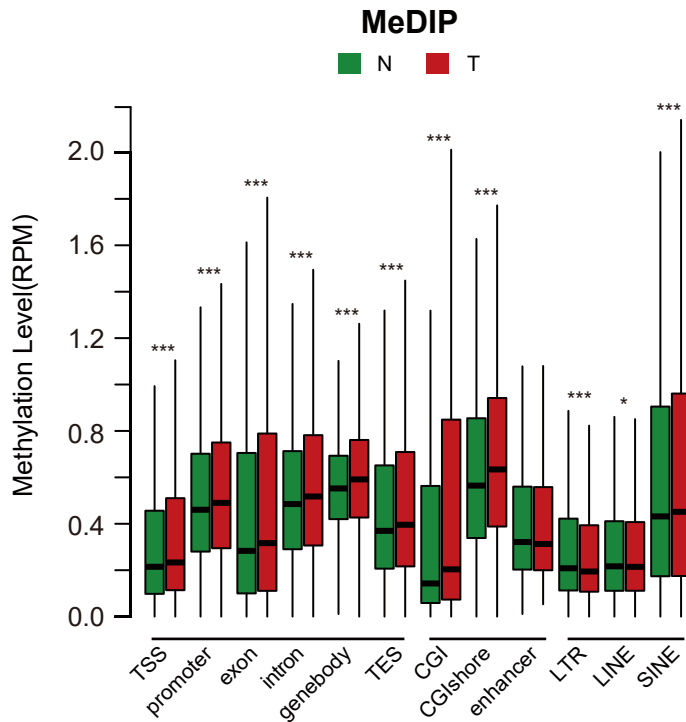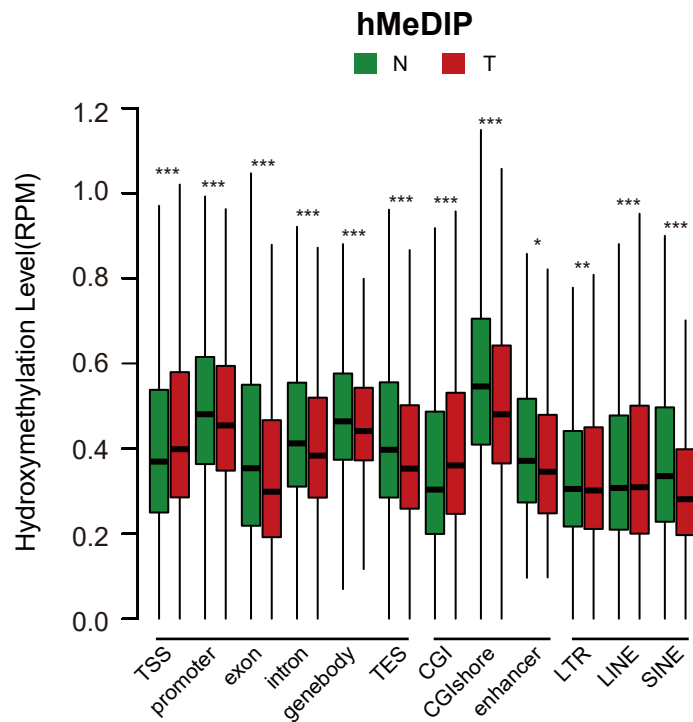

Supplement: Supplementary file 4 — Figure S3. Methylation and hydroxymethylation levels distribution in different types of genomic elements. Normal is shown by green, and tumor is shown by red. TSS: transcriptional start sites; TES: transcriptional end sites; CGI: CpG islands; LTR: long terminal repeat; SINE: short interspersed nuclear elements; LINE: long interspersed nuclear elements. *, P < 0.05, **, P < 0.01 and ***, P < 0.001. (PDF 388 kb) [file 13148_2018_458_MOESM4_ESM.pdf]

## Correlation between mC and hmC in N

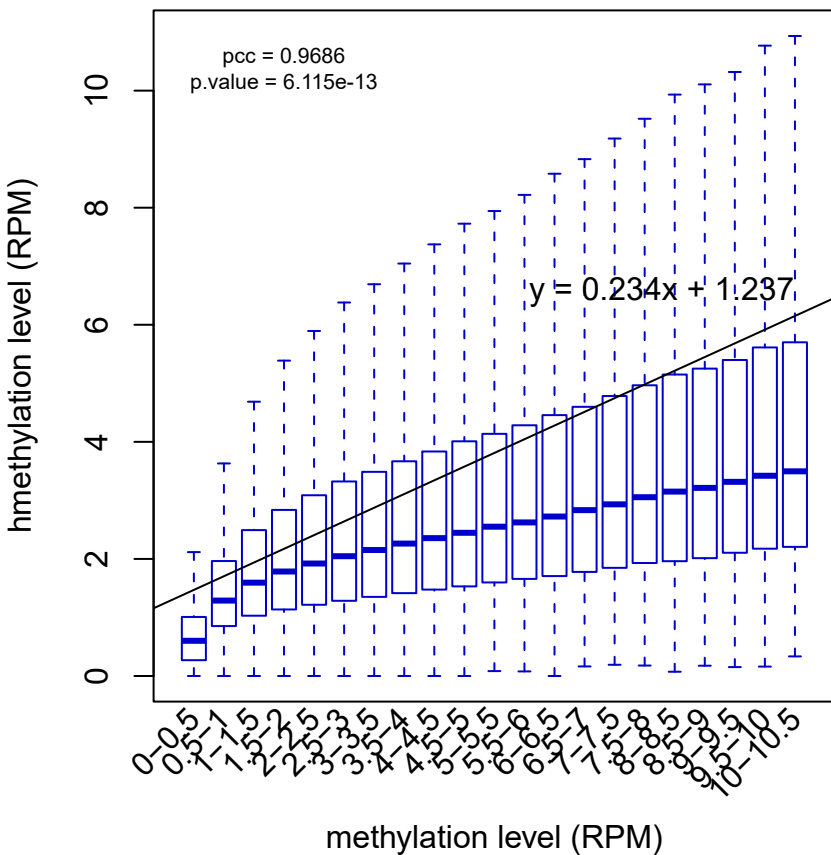

## Correlation between mC and hmC in T

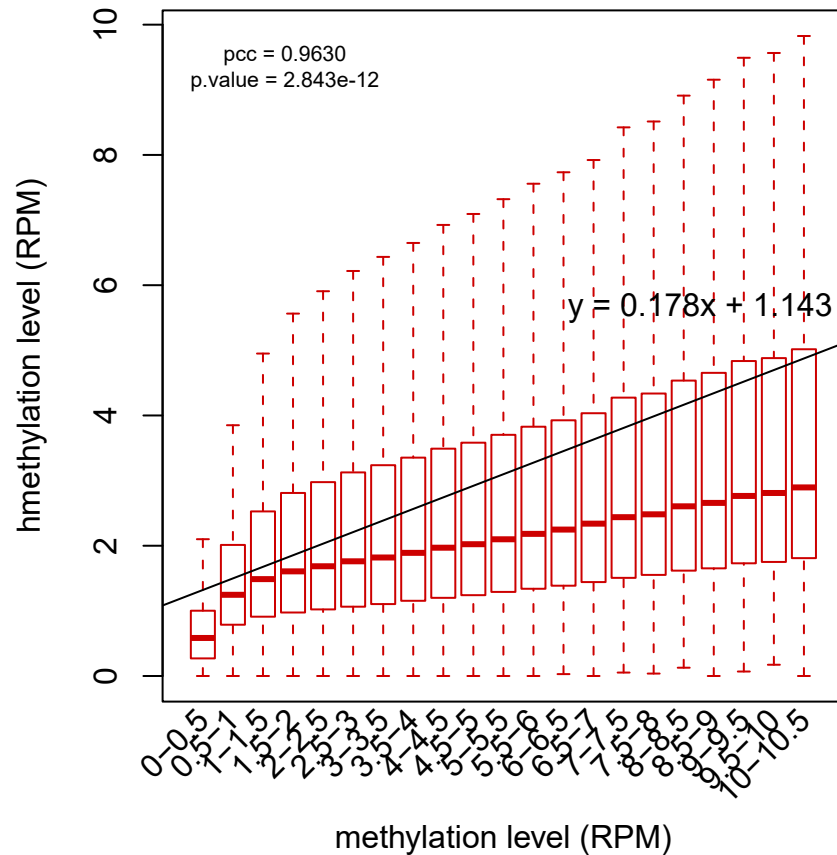

Supplement: Supplementary file 5 — Figure S4. Correlation between 5-mC and 5-hmC in normal (left) and tumor (right) samples. The whole genome was divided into 0.5 kb windows and the levels of 5-mC and 5-hmC were classified into different groups according to the RPMs of Medip and hMedip, respectively. (PDF 172 kb) [file 13148_2018_458_MOESM5_ESM.pdf]

**Medip**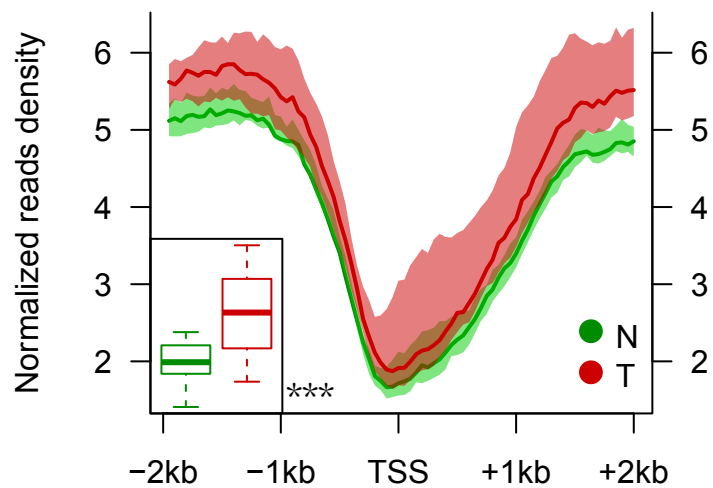**hMedip**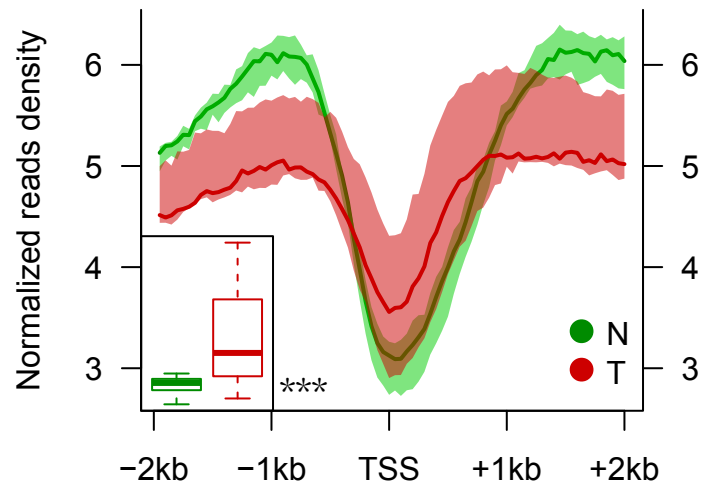**Medip**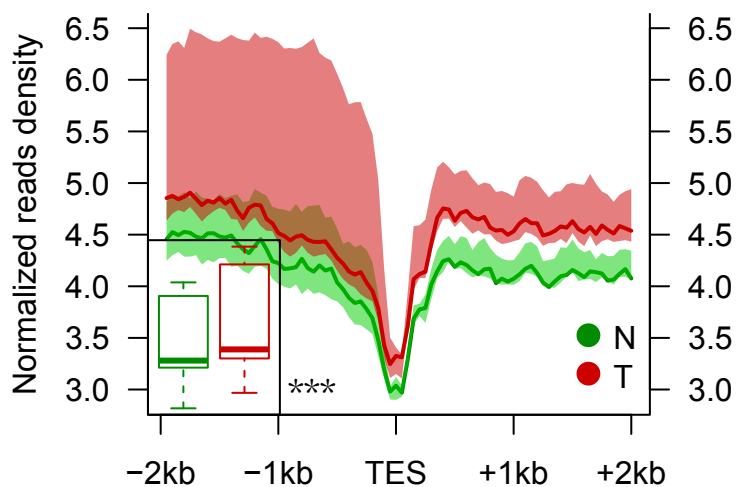**hMedip**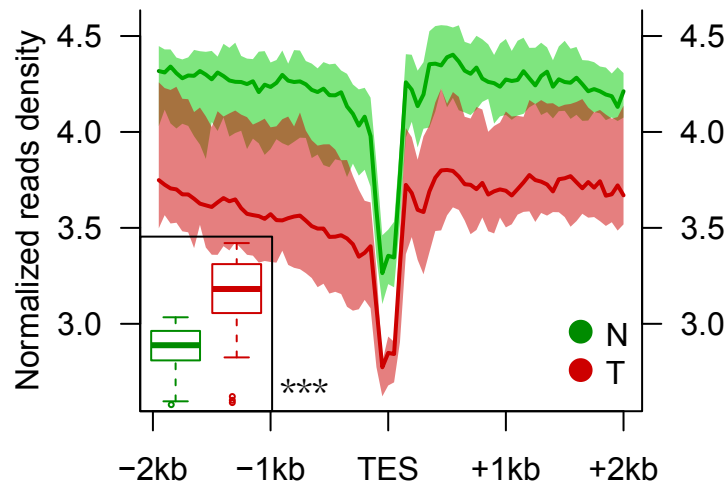

Supplement: Supplementary file 6 — Figure S5. Mean profiles with standard deviation (SD) over TSS and TES regions. 4-kb regions were divided in 80 bins from 5′ to 3′ end, and the mean RPM values (with SD) within each bin for each modification type was determined (5-mC left, 5-hmC right). Additionally, SD distribution are be shown at bottom left in each region. (PDF 217 kb) [file 13148_2018_458_MOESM6_ESM.pdf]

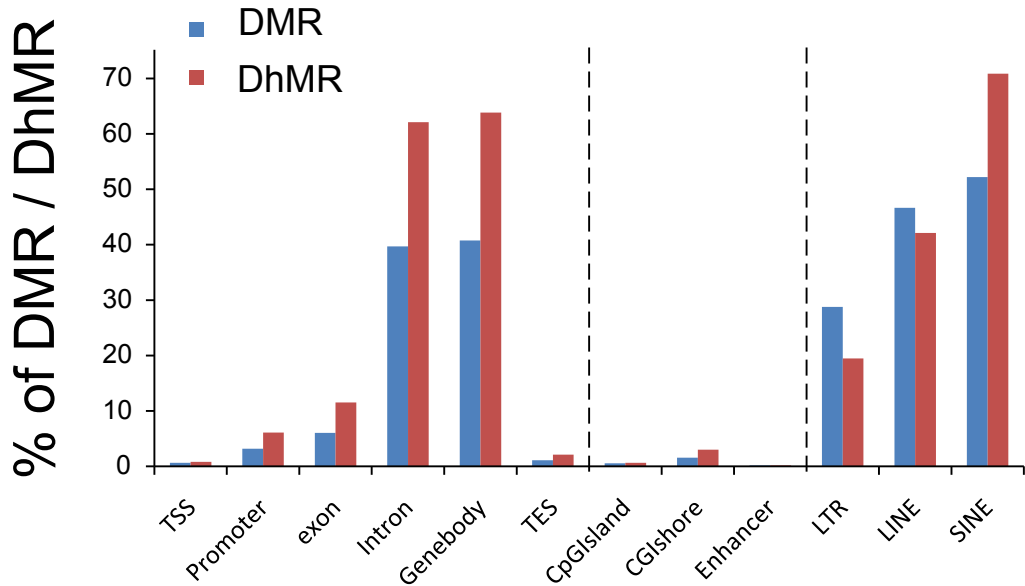

Supplement: Supplementary file 7 — Figure S6. Distribution of DMRs and DhMRs in different types of genomic elements. (PDF 162 kb) [file 13148_2018_458_MOESM7_ESM.pdf]

26,440 kb

26,520 kb

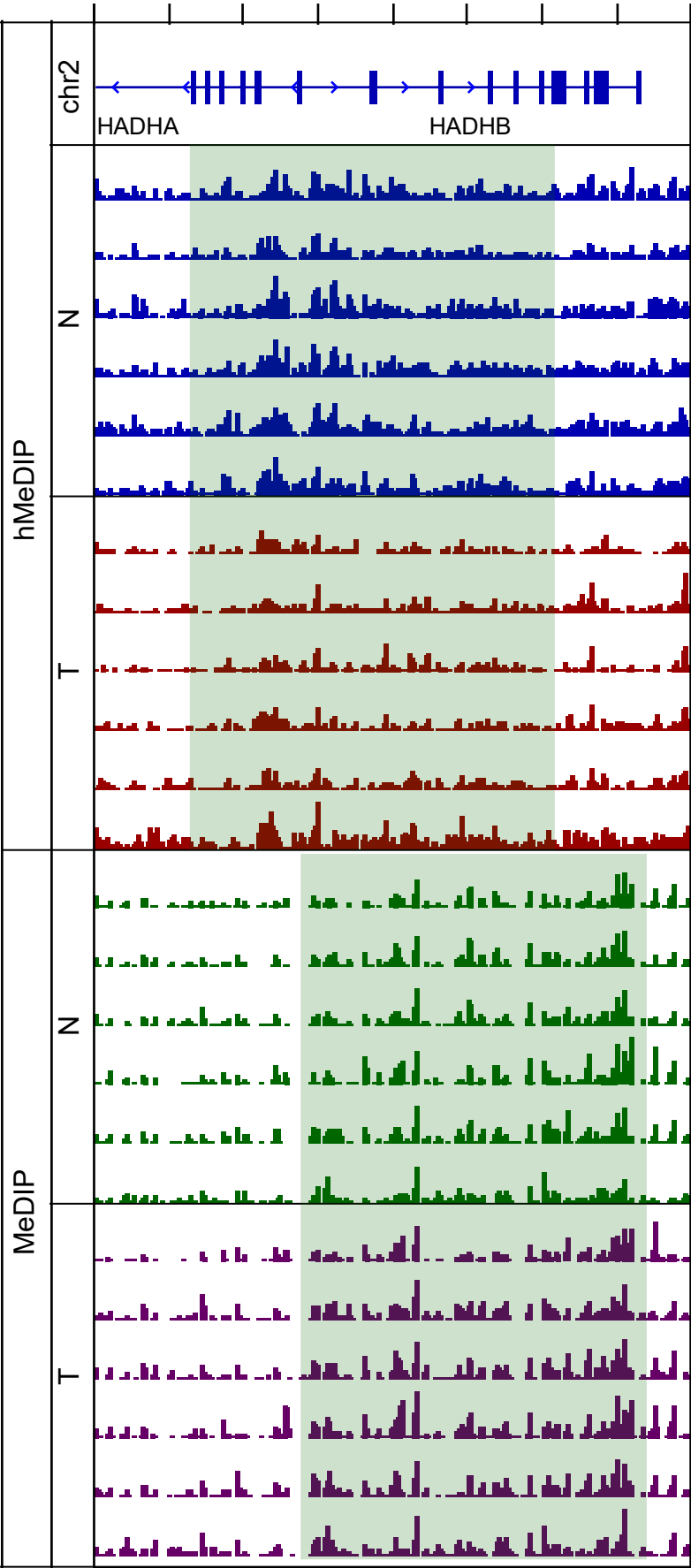

Supplement: Supplementary file 9 — Figure S8. Visualization of DMRs (bottom) and DhMRs (top) in HADHB. DMR and DhMR are denoted by the green box. (PDF 231 kb) [file 13148_2018_458_MOESM9_ESM.pdf]
