# Supplementary material for: Integrated analyses of multi-omics reveal global patterns of methylation and hydroxymethylation and screen the tumor suppressive roles of HADHB in colorectal cancer
Source: Clin Epigenetics. 2018 Mar 2;10:30. doi: 10.1186/s13148-018-0458-3 (PMC5833094; doi:10.1186/s13148-018-0458-3)
Supplement: Supplementary file 8 — Figure S7. Gene functional pathways of DMR, DhMR and DEG. Three pathway, metabolic pathways, purine metabolism and axon guidance, overlapped pathways among DMR, DhMR and DEG. (PDF 138 kb) [file 13148_2018_458_MOESM8_ESM.pdf]

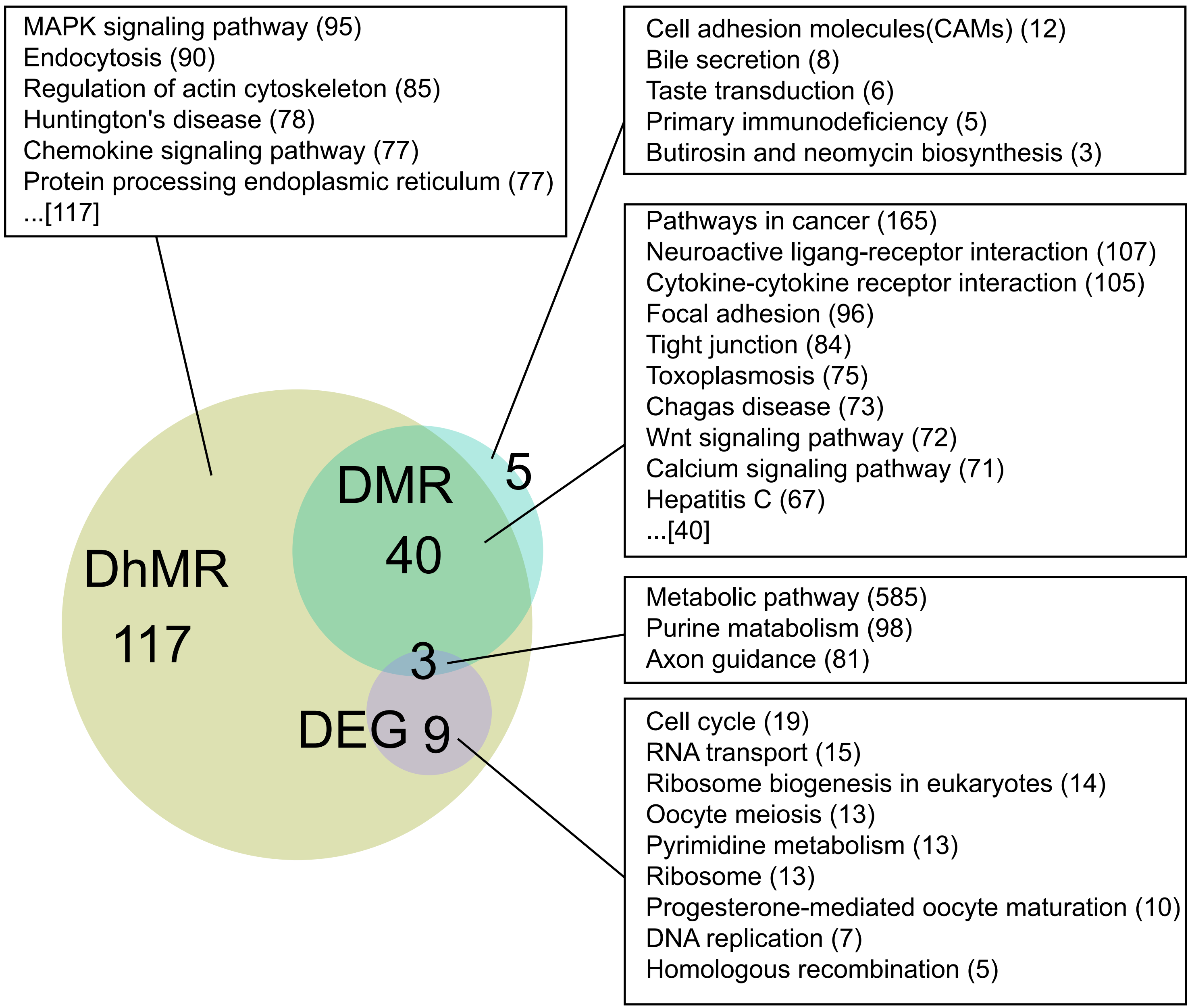
MAPK signaling pathway (95)
Endocytosis (90)
Regulation of actin cytoskeleton (85)
Huntington's disease (78)
Chemokine signaling pathway (77)
Protein processing endoplasmic reticulum (77)
...[117]
Cell adhesion molecules(CAMs) (12)
Bile secretion (8)
Taste transduction (6)
Primary immunodeficiency (5)
Butirosin and neomycin biosynthesis (3)
Pathways in cancer (165)
Neuroactive ligang-receptor interaction (107)
Cytokine-cytokine receptor interaction (105)
Focal adhesion (96)
Tight junction (84)
Toxoplasmosis (75)
Chagas disease (73)
Wnt signaling pathway (72)
Calcium signaling pathway (71)
Hepatitis C (67)
...[40]
Metabolic pathway (585)
Purine matabolism (98)
Axon guidance (81)
Cell cycle (19)
RNA transport (15)
Ribosome biogenesis in eukaryotes (14)
Oocyte meiosis (13)
Pyrimidine metabolism (13)
Ribosome (13)
Progesterone-mediated oocyte maturation (10)
DNA replication (7)
Homologous recombination (5)
DMR
5
40
DhMR
117
3
DEG
9
